# On the Future Perspectives of Some Medicinal Plants within *Lamiaceae* Botanic Family Regarding Their Comprehensive Properties and Resistance against Biotic and Abiotic Stresses

**DOI:** 10.3390/genes14050955

**Published:** 2023-04-22

**Authors:** Dan Ioan Avasiloaiei, Mariana Calara, Petre Marian Brezeanu, Otilia Cristina Murariu, Creola Brezeanu

**Affiliations:** 1 Vegetable Research and Development Station, 600388 Bacău, Romaniacalaramariana@gmail.com (M.C.); 2 Department of Food Technology, Iasi University of Life Sciences (IULS), 700490 Iasi, Romania; otiliamurariu@uaiasi.ro

**Keywords:** aromatics, nutrient content, essential oils, salinity stress, drought stress, breeding prospects

## Abstract

*Lamiaceae* is one of the largest botanical families, encompassing over 6000 species that include a variety of aromatic and medicinal spices. The current study is focused on three plants within this botanical family: basil (*Ocimum basilicum* L.), thyme (*Thymus vulgaris* L.), and summer savory (*Satureja hortensis* L.). These three species contain primary and secondary metabolites such as phenolic and flavonoid compounds, fatty acids, antioxidants, and essential oils and have traditionally been used for flavoring, food preservation, and medicinal purposes. The goal of this study is to provide an overview of the nutraceutical, therapeutic, antioxidant, and antibacterial key features of these three aromatics to explore new breeding challenges and opportunities for varietal development. In this context, a literature search has been performed to describe the phytochemical profile of both primary and secondary metabolites and their pharmacological uses, as well as to further explore accession availability in the medicine industry and also to emphasize their bioactive roles in plant ecology and biotic and abiotic stress adaptability. The aim of this review is to explore future perspectives on the development of new, highly valuable basil, summer savory, and thyme cultivars. The findings of the current review emphasize the importance of identifying the key compounds and genes involved in stress resistance that can also provide valuable insights for further improvement of these important medicinal plants.

## 1. Introduction

The economic importance of medicinal and aromatic plants within the context of agro-alimentary, pharmaceutical, natural cosmetics, and perfume development uses is of paramount significance. In addition to providing food flavoring and pleasing aromas, their secondary metabolites and antioxidants provide additional nutritional value and make them an invaluable part of the human diet, alongside cereals, fruits, and vegetables [1,2].

The *Lamiaceae* botanical family encompasses around 236 genera and over 6000 species of herbs and shrubs that have a global distribution. Within this family, basil (*O. basilicum* L.), thyme (*T. vulgaris* L.), and summer savory (*S. hortensis* L.) are species of particular importance, both for their specialized metabolites, such as essential oils and various non-volatile constituents with multiple applications in the food industry, cosmetics, and medicine, and their amazing adaptability to a whole range of biotic and abiotic stress factors. They are highly valued for their nutritional, medicinal, and industrial properties, providing flavor and fragrance to food, promoting health and well-being in traditional medicine due to the anti-inflammatory, antiseptic, and analgesic properties of their essential oils, and serving as valuable ingredients in various industries (perfumes, cosmetics, and cleaning agents) [1,2].

With regards to the main abiotic stress factors, the climate change evidence from the past decade indicates that salinity and drought will likely be the highest causes of significant concern. Salinity has emerged as a major environmental factor that has impacted over 20% of cultivated land worldwide, with the affected regions continuing to expand every year [3,4]. In fact, there is a significant risk that salinization may affect more than 50% of arable land by the mid-21st century [5], posing a serious challenge to global food security. Salinity can have a negative impact on *Lamiaceae* aromatic plants, affecting their growth, yield, and quality. High levels of salt in the soil decrease water availability and impose osmotic stress conditions on plants, leading to reduced photosynthesis and yield limitation [3]. In response to such conditions, the uptake and transport of the major essential ions (such as Ca, K, Mg, and nitrate) is transiently decreasing, and as a consequence, it impacts the amount and quality of secondary metabolites, such as essential oils. There is a significant increase in the reactive oxygen species (ROS) production under osmotic stress conditions that can exceed the scavenging ability of the plants. The accumulation of ROS has harmful cellular effects, such as DNA damage, membrane lipid peroxidation, and enzymatic activity impairment.

Drought stress is another significant environmental concern, representing a serious threat to agriculture worldwide by reducing the yield and quality for principal cash crops. It may have a negative impact on *Lamiaceae* aromatic plants as well affecting their growth, yield, and quality. However, most of these plants possess the ability to respond to drought stress at morphological, anatomical, physiological, biochemical, and molecular levels with a series of adjustments, allowing the plant to avoid the stress or to increase its tolerance [6,7].

## 2. Methods

Plenty of scientific literature discusses the benefits of these three aromatic species, and there is an abundance of data regarding their adaptation to key stressors and breeding perspectives. As a result, we searched SCOPUS and the Google Academic database for topics relating to “*O. basilicum*”, “*T. vulgaris*”, and “*S. hortensis*” and performed an extensive keyword search for “(Aromatic plant species name) composition and properties” and ”(Aromatic plant species name) (a) biotic stress resistance.” Our search generated around 350 documents published over a period of 35 years (1989–2022). The returned results highlighted a multitude of studies from countries with a long-standing traditional cultivation of these plants, especially from the Middle and Far East, focusing on a variety of complex topics. 

This review aims to present the progress that has been made in studying the resistance potential of these three species on a series of stressors and to emphasize the need to focus on breeding and developing new cultivars with higher resistance capacity.

## 3. Discussion

### 3.1. Aromatic Plant Composition and Accessions Availability

A synthetic characterization of the main biocomponents of the three species studied, including the volatile oils, is presented in Table 1.

### 3.2. O. basilicum L. Row Plant and Essential Oil Composition and Accession Availability

*Ocimum* is one of the largest genera in the *Lamiaceae* family, which consists of 65 species native to Africa, South America, and Asia [23]. Among the species, sweet basil (*O. basilicum* Linn.), which originated from the warm tropical climates of India, Africa, and southern Asia, is probably the most important crop, being cultivated as a culinary herb worldwide under various ecological circumstances [24]. Both the raw plant and essential oil of basil have marked culinary, pharmaceutical, and cosmetic purposes [23]. It is used frequently in traditional medicine, having antispasmodic, stomachic, carminative, anti-ulcerogenic, anti-inflammatory, anti-carcinogenic, analgesic, stimulant radioprotective, and febrifuge properties [25]. Basil’s leafy components exhibit antimicrobial properties and antioxidant activity [26,27] that can be used for alleviating pain and otitis [28]. The essential oil extracted from European genotype basil is recognized for its superior aroma, primarily comprising linalool and methyl chavicol.

Due to their vast diversity, various plant species and cultivars exhibit unique levels of resistance and ability to withstand physiological functions and produce yields in varying environmental conditions and stressful situations. 

**Figure 1:**
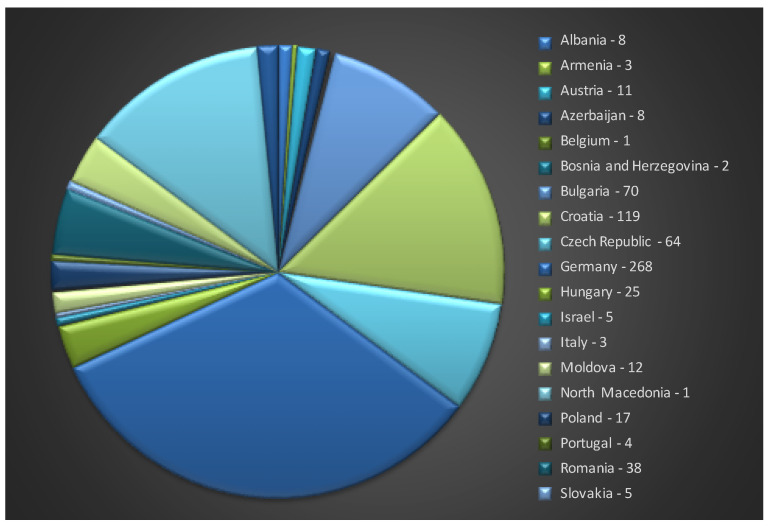
*O. basilicum* L. accession availability at the European level (https://eurisco.ipk-gatersleben.de/apex/eurisco_ws/r/eurisco/taxon-search-results?p26_genus=OCIMUM&p26_species=BASILICUM, accessed on 14 February 2023).

Furthermore, at the European level, based on the EURSICO National Inventory Report Taxonomy (ipk-gatersleben.de), a total number of 834 *O. basilicum* L. accessions are available for multiplication and future breeding perspectives (Figure 1). The countries that have uploaded the largest number of accessions are Germany (268 acc.), Croatia (119), the Czech Republic (64), and Romania (38).

### 3.3. T. vulgaris L. Composition and Accession Availability

The *Thymus* genera of the *Lamiaceae* family is highly significant due to its large number of species [29]. Thyme (*T. vulgaris* L.), commonly known as garden thyme or common thyme, appears in many different areas worldwide, including the drier Mediterranean regions [30], and is the most commercially cultivated species in the genus *Thymus* [31] due to the dietary trends in the recent decades [32]. It has several aromatic and medicinal properties. The leaves can be used either fresh or dried as a flavoring component in various culinary preparations, containing a high ratio of minerals (K, Ca, Mg, Fe, Mn, and Se), antioxidants (flavonoids, phenolic compounds such as pigenin, naringenin, luteolin, thymonin, lutein, and zeaxanthin), and vitamins (A, B6, B9, C, E, and K) [7,33,34]. In addition, the essential oil of common thyme is extracted by the distillation of the fresh leaves and flowering tops, and it contains 20–54% thymol, which is a monoterpene known as the main active ingredient with a wide range of pharmacological properties [7,35,36]. It has demonstrated anti-pathogenic and antioxidant effects, being intensively utilized in various industries, particularly in medication, agriculture, and food production.

**Figure 2:**
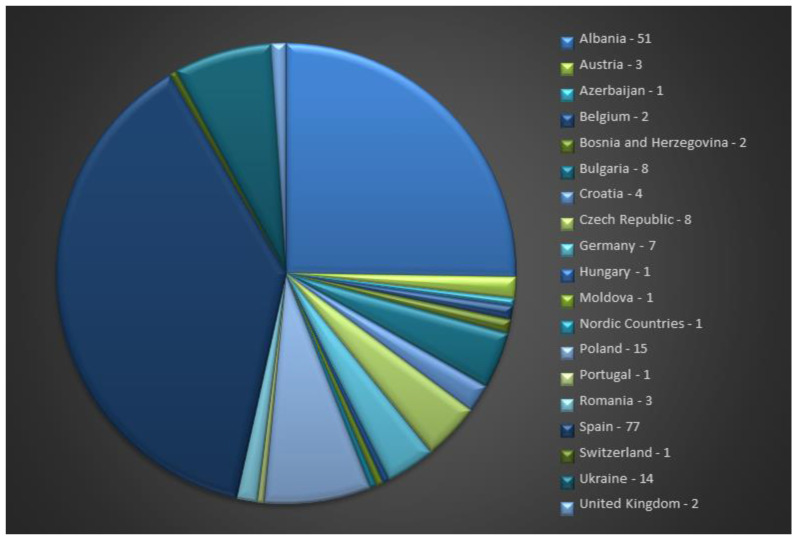
*T. vulgaris* L. accession availability at the European level (https://eurisco.ipk-gatersleben.de/apex/eurisco_ws/r/eurisco/taxon-search-results?p26_genus=THYMUS&p26_species=VULGARIS, accessed on 14 February 2023 ).

At the European level, the total number of *T. vulgaris* L. accessions is 202, with Spain (77), Albania (51), Poland (15), and Ukraine (14) having uploaded the highest numbers of accessions to the EURISCO database (Figure 2).

### 3.4. S. hortensis L. Composition and Accession Availability

Summer savory (*S. hortensis* L.) is an annual herbaceous plant that is among the principal *Satureja* species grown in southern Europe, as well as central and southwestern Asia [37]. It contains numerous vitamins, including B-complex vitamins, vitamin A, vitamin C, niacin, thiamine, and pyridoxine, as well as carvacrol, terpinene, cymene, and caryophyllene, which make it an excellent choice for medicinal purposes [19,38]. The aerial parts of the *Satureja* species, such as *S. hortensis*, contain essential oil that is widely used in the medicine, food, and health industries for therapeutic purposes. Scientific studies have highlighted several pharmacological properties of *Satureja*, such as antispasmodic, antioxidant, antimicrobial, antidiarrheal, and sedative properties [37,39,40,41]. Its beneficial effects on hypertension have also been discussed [42].

The savory essential oil contains two major compounds, thyme and carvacrol, that have antiseptic, antifungal, and antibacterial properties [3]. The concentration and composition of secondary metabolites in savory oils, such as y-terpinene, p-cymene, carvacrol methyl ether, and caryophyllene, play significant ecological roles as they possess insecticidal, antifungal, and antibacterial properties [43,44,45].

**Figure 3:**
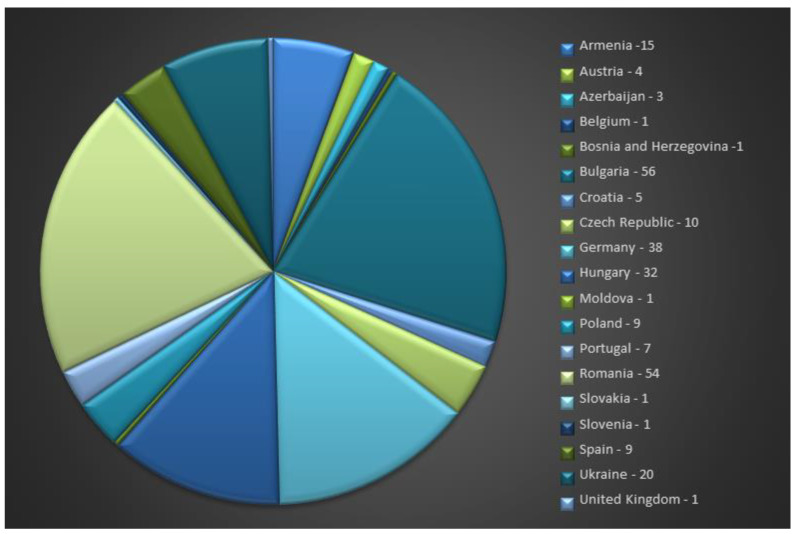
*S. hortensis* L. accession availability at the European level (https://eurisco.ipk-gatersleben.de/apex/eurisco_ws/r/eurisco/taxon-search-results?p26_genus=SATUREJA&p26_species=HORTENSIS accessed on 14 February 2023).

At the European level, the total number of *S. hortensis* L. accessions is 269, with Bulgaria (56), Romania (54), Germany (38), and Hungary (32) being the countries with the highest number of accessions uploaded to the EURISCO database (Figure 3).

### 3.5. Aromatic Plant Biological Activities and Stress Resistance

Various elements, such as genetic and ecological factors, have a notable impact on the chemical components of medicinal plants and their physiological and morphological characteristics [46]. Diverse environmental circumstances can be the primary cause of variability in morphological characteristics, which may induce alterations in the phenotype in the short term and in its genotype in the long term [47]. The presence of abiotic environmental stressors, such as salinity and drought, can inhibit the plant growth and development [48,49]. 

Essential oils perform a crucial function in plant defense by serving as antiviral, antibacterial, antimycotic, and insecticidal agents and deterring herbivores [50]. Due to these properties, diverse plant essential oils may serve as remedial or auxiliary agents in the pharmaceutical sector [51] and function as fragrances, seasonings, and natural preservatives in the food industry [52]. Finally, these oils may also serve as eco-friendly and biodegradable substances for protecting plants in agriculture [53].

### 3.6. O. basilicum L.—Biological Activities and Stress Resistance

Basil is regarded as a functional food plant due to its abundant secondary metabolites and antioxidant traits that are believed to enable oxidative stress disease prevention. The constituents of basil’s essential oil (EO) are known to differ significantly based on the genetic factors (cultivar, origin, season, chemotype, and phonological stage), environmental factors (climatic conditions, agricultural practices, and postharvest processes), and the combined effects of these factors [4,54]. Oxygenated monoterpenes and phenylpropanoids are the primary chemical compounds present in the *Ocimum* genus, while linalool, eugenol, methyl chavicol, methyl cinnamate, methyl eugenol, and geraniol are some of the significant constituents identified in various *O. basilicum* cultivars and chemotypes [55]. These constituents act as potent antioxidants by scavenging free radicals and functioning as electron donors, making them effective in safeguarding the plants against pathogens and predators. At the cellular level, they can also protect cells from the adverse effects of ROS arising from different abiotic stressors [56].

Plants that have a high concentration of antioxidants can resist damage caused by ROS. These antioxidants can also function as protective substances [57,58]. The existence of phenolic compounds relies on various factors, such as the type of soil, plant species, genetics, growth stage, and location [59]. The primary phenolic compounds responsible for the antioxidant effects in basil are caffeic acid (CA) and rosmarinic acid (RA), which is an ester of CA. These compounds are mostly produced in roots and leaves [60]. CA is an active participant in plant physiology and stress tolerance mechanisms [56]. RA has been utilized as an anti-inflammatory, anti-proliferative, and chemoprotective agent [61]. The synthesis of RA in plants occurs through the phenylpropanoid and tyrosine-derived pathways. Phenylalanine ammonia-lyase (PAL) is the principal enzyme in the phenylpropanoid pathway, catalyzing the transformation of l-phenylalanine to trans-cinnamic acid and ammonia. Research has confirmed that PAL plays a role in RA biosynthesis [62]. An increase in PAL activity induced by stress could be the starting point for the cells to adapt to drought conditions [63]. Phosphorus (P) is an essential element to produce secondary metabolites in plants, and its availability can affect the amount and composition of phenolic compounds. A competition exists between the production of phenolic compounds and the required proteins for growth. 

Regarding the response of *O. basilicum* L. to different stressors, Table 2 presents the main anatomical, physiological, and molecular changes, as well as some contributing factors that could occur. 

Salinity stress has various negative impacts on plants, including reduced growth and water content [77]. Additionally, salinity stress results in reduced soil moisture content and limited water absorption from the soil, leading to osmotic stress in plants [66]. However, the osmotic potential significantly improves. Moreover, in many plants, salt stress increases the levels of cell free radicals to a point where it can damage the membrane, further intensifying the effects of the stress. The sensitivity of plants to salt stress can be measured by malondialdehyde (MDA), which is a widely used parameter for estimating lipid peroxidation in plant tissue that increases under oxidative stress. At the cellular level, osmotic stress modifies the properties and composition of the membrane lipids. MDA accumulation increased in the severity of NaCl stress in the leaves of summer savory (*S. hortensis* L.) and *O. basilicum* L. The MDA content serves as an indicator of oxidative stress resulting from membrane lipid peroxidation, and it can be reduced by lowering lipid peroxidation and increasing the activity of antioxidant enzymes in salt-affected plants [64].

The composition and amount of essential oils can be influenced by environmental factors, including salinity stress, which can have a negative impact on plant growth and osmotic balance [78]. Under salinity stress, proline plays a crucial role in osmotic adjustment, helping to maintain the osmotic balance of the plant and enhance its tolerance to salt stress [79]. Proline also acts as an ROS scavenger, protein stabilizer, and osmo-protectant [80]. The accumulation of proline during drought stress has been shown to be related to improved plant performance, probably due to its antioxidant properties and its ability to stabilize macromolecules [81,82].

Regarding the drought stress, the accumulation of secondary metabolites is a defensive mechanism employed by plants to cope with stress by altering their cellular metabolism to overcome various challenges [83].

The concentration of leaf chlorophyll (Ch) is a crucial physiological characteristic that directly impacts a plant’s photosynthetic ability. Both chlorophyll-a and chlorophyll-b levels in sweet basil plants decreased when they were deprived of water. The amount of chlorophyll in leaves, which is an indicator of plant vigor, is influenced by a variety of environmental factors. The reduction in chlorophyll content due to the water scarcity could be linked to the generation of ROS in cells [84], which have negative effects on plants in stressful situations. The plant water status is an indicator of their response to water scarcity, with higher relative water contents indicating a healthy plant condition [85]. In experiments involving various water supplies, the accurate evaluation of the plant water status is crucial. The water content of plants can be expressed per unit of fresh or dry weight (or, less commonly, per unit of leaf area) [86]. Particularly, the leaf relative water content (RWC) is utilized as a dependable measure of a plant’s susceptibility to dehydration [87]. To reduce water loss through the leaves, the transpiration rate can be adjusted, and the leaf area can be limited. Typically, plants restrict water loss through their foliage by closing stomata, which reduces the rate of transpiration from the leaves. Nevertheless, under conditions of water scarcity, the root water uptake may be more critical in mitigating the damage caused by drought stress than controlling water loss through the leaves [88].

Employing plant growth regulators (PGRs) represents a viable means of enhancing plant resilience to stress, alongside other techniques such as selective breeding and genetic modification. There are numerous compounds that can alleviate drought-related stress in plants [89]. Salicylic acid, or 2-hydroxybenzoic acid, is a phenolic compound with hormone-like properties that can disrupt plant growth regulation, particularly when confronted with diverse stresses [90]. Additionally, it may trigger various physiological and biochemical functions in plants.

Moreover, salicylic acid (SA) has the ability to hinder catalase (CAT) function, which could result in the accumulation of hydrogen peroxide (H_2_O_2_). This, in turn, may stimulate the operation of ROS-detoxifying enzymes and the production of antioxidant metabolites [91].

The ability of plants to adapt to environmental pressures, such as drought or salinity, requires temporary and long-lasting reductions in transpiration water flow. This physiological reaction is influenced by both inherent and induced genetic factors [92]. Stomata are of paramount importance in transpiration management, regulating plant water loss through density on the leaf surface and the mechanism of closure in response to environmental stimuli.

On the biotic nature stressors, mites prevent harm to the epidermis, thus reducing the chances of the leaf surface detecting the attack and postponing the plant’s reaction. This pressure results in a modification of the plasma membrane potential and consequent alterations in the concentration of free cytosolic Ca^2+^, which set off a signal that initiates a series of reactions [93]. One of the primary effects of biotic stress encountered by the plant is a rise in the cellular levels of ROS, which are then transformed into H_2_O_2_.

### 3.7. T. vulgaris L.—Biological Activities and Stress Resistance

*T. vulgaris* L. displays antimicrobial, anti-inflammatory, antioxidant, and immunomodulatory properties, being effective against a variety of ailments related to the respiratory, cardiovascular, and nervous systems, among others [94,95]. These effects are ascribed to phenolic acids, other phenols, and particularly the plant’s essential oil. The essential oil is composed mainly of thymol, carvacrol, geraniol, α-terpineol, 4-thujanol, linalool, 1,8-cineole, myrcene, γ-terpinene, and p-cymene. The key components’ abundance varies greatly depending on the plant chemotype, with the thymol chemotype being the most widespread [96,97]. This versatile plant finds widespread use in the food and pharmaceutical industries [51,52]. It is also a promising agent for crop protection and storage preservation [52]. From an economic perspective, the plant’s biomass yield and essential oil quality are crucial, and various factors can affect them, including crop nutrition, manure application, water stress, seasonal variations, or processing [98,99]. 

When using the DPPH free radical scavenging method, *T. vulgaris* demonstrated a robust antioxidant activity of approximately 85% [100,101,102]. A similar outcome was observed regarding the antioxidant activity of the thyme methanol extract [103].

The disk diffusion method revealed that the extract exhibited very strong inhibition (20 mm inhibition zone) against various bacteria, including *Bacillus subtilis*, *Staphylococcus aureus*, *Pseudomonas aeruginosa*, and *Candida albicans* [104]. However, its inhibitory activity against *Candida tropicalis* was found to be moderate [13].

The antimicrobial activity of essential oils (EOs) is dependent on their chemical constituents. The antimicrobial activity of the analyzed EO is likely associated with the presence of phenolic compounds (thymol) and terpene hydrocarbons (γ-terpinene) [105,106]. It is believed that p-Cymene, the third major component, has synergistic effects with thymol and γ-terpinene [107], which may contribute to the observed antimicrobial activity. Furthermore, several studies have indicated that EOs exhibit stronger antimicrobial activity than their major constituents or mixtures [108,109], suggesting that minor components may have synergistic effects and emphasizing the importance of all components in relation to the biological activity of EOs [13].

The *T. vulgaris* essential oil demonstrated potent antimicrobial and antibiofilm effects, with MIC values ranging from 0.0625% to 2% *v*/*v* [110]. It also exhibited lower minimum inhibitory concentration values compared with the antibiotics tested on eradicating *Candida* genus biofilm [111]. When utilized in the vapor phase, it could serve as a viable alternative to antimicrobials in the food industry due to the lower concentration of EO required compared with the liquid phase contact effect [112]. 

The thyme essential oil contains elevated levels of TPC along with strong radical scavenging ability against DPPH, ABTS, and linoleic acid radicals, in addition to iron chelating capabilities. This positive correlation highlights the antioxidant potential of TEO to combat various oxidation systems and prevent oxidative damage [113].

At a concentration of 0.1 mg/mL, thyme oil and a CNC-based formulation of thyme white oil demonstrated complete larvicidal activity against *Aedes albopictus* [114].

The composition of bioactive secondary metabolites synthesized by medicinal plants varies widely depending on the species, having a profound impact on their relationship with endophytic microorganisms [115]. *Bacillus* spp. connected with *T. vulgaris*, such as *Bacillus sonorensis* (EGY05), *Bacillus tequilensis* (EGY21), and *Bacillus mojavensis* (EGY25), generated plant-growth-stimulating substances such as auxin, fixed nitrogen, soluble phosphate and iron, and lytic enzymes such as chitinase, cellulase, protease, and lipase. These bacteria may provide novel tactics to alleviate salt stress [116].

Phenolic substances and their corresponding enzymes (PAL and PPO) could potentially serve as protective factors against drought stress [6] that impacts the water status of leaves, pigments, and stomatal conductance, leading to the inhibition of photosynthesis [6]. The PPOs, in particular, play a role in various processes such as the Mehler reaction, the photoreduction of molecular oxygen by PSI, the regulation of oxygen levels in plastids, and the generation of the phenylpropanoid pathway [117]. The decrease in chlorophyll-a, chlorophyll-b, and total chlorophyll concentrations on thyme plants experiencing water stress may be viewed as an important regulatory measure to prevent excessive light absorption and to restrict the over-reduction of the photosynthetic electron transport chain, thereby limiting the generation of ROS [118]. ROS have been shown to be produced by both biotic and abiotic stresses [119], and these molecules are responsible for most of the oxidative damage to biological structures, including DNA, RNA, amino acids, proteins, and lipids [120].

Hydrogen peroxide (H_2_O_2_) is one of the most stable ROS which is produced in plant cells during different physiological processes, including photosynthesis, photorespiration, and, to a relatively lesser extent, respiration; it plays an important role as a signaling molecule under stressful conditions [121].

Malondialdehyde (MDA) is also widely known as a biochemical marker to increase the activity of ROS and the oxidative stress in plant tissues under adverse conditions. It is considered the most final product of lipid peroxidation and an important indicator of the oxidative damage that could occur in the cellular membrane under different stress conditions [7].

In plants, mutations on the epigenetic regulator histone deacetylase-6 (HDA-6) appear to improve survival in drought conditions [122]. This response is associated with the expression of genes involved in acetic acid biosynthesis. Therefore, in conditions of water stress, there would be a relationship between HAD-6 and the regulation of genes involved in acetic acid synthesis [123].

Preserving the integrity of cellular membranes during stressful circumstances is deemed a crucial aspect of any salinity adaptation mechanisms. The percentage of electrolyte leakage (ELP) indicates the level of injury to cell membranes. The application of salicylic acid (SA) amplified the ion leakage in thyme seedlings exposed to salt stress at greater concentrations, indicating that SA concentrations play a crucial role in saline environments. 

The potential of ascorbic acid in mitigating and modifying the effects of salt stress on plants is well known. As a rule, its concentration is higher in leaves compared with other plant parts and is 5–10 times higher than that of glutathione [124]. In addition, the ascorbic acid’s antioxidant role has been confirmed [125]. Therefore, plants require high endogenous levels of ascorbic acid to regulate various processes of plant metabolism in addition to countering oxidative stress. Endogenous levels of ascorbic acid can be elevated by exogenously administering ascorbic acid via the rooting medium, as a foliar spray or as seed priming. It plays a significant role in photosynthesis, specifically by regulating the redox state of photosynthetic electron carriers through the Mehler peroxidase reaction with ascorbate peroxidase and acting as a co-factor for violaxanthin deep oxidase, which is involved in xanthophyll cycle-mediated photoprotection [126]. As a result, in plants treated with ascorbic acid, high levels of pigments can work synergistically with the ascorbic acid to provide an efficient barrier against oxidation under salinity stress. Ascorbic acid can mitigate the detrimental effects of salinity by increasing the auxin and gibberellin content while reducing abscisic acid levels [127], which may help protect the photosynthetic apparatus and subsequently increase photosynthetic pigments.

During stressful periods, the accumulation of compatible osmolytes, such as proline, can serve as an appropriate marker for heavy metal contamination. In addition, proline may exhibit antioxidative properties that safeguard the cells from the detrimental effects of ROSs induced by Cd contamination due to a conducive environment for Cd sequestration and phytochelatin synthesis [128].

The main anatomical, physiological, and molecular changes of *T. vulgaris* related to different stressors are presented in Table 3.

### 3.8. S. hortensis L.—Biological Activities and Stress Resistance

The EO extracted from summer savory contains a significant amount of carvacrol, which plays a crucial role in various biological activities, such as antimicrobial, antioxidant, antidiabetic, antihyperlipidemic, antispasmodic, antinociceptive, anti-inflammatory, antiproliferative, sedative, and reproduction stimulatory effects [145]. The EO content in different species of this genus is more than 5%, the major oil constituents being carvacrol, thymol, γ-terpinene, and borneol [146]. The chemical composition of the plant extracts is influenced by several factors, including the plant part used, harvest time, extraction method, plant cultivar or genotype, geographical location, storage, and climatic conditions [39].

Regarding its antimicrobial properties, summer savory volatile oils exhibit actions on cell membranes, causing interference, destabilization, and consequent effects on the phospholipid bilayer and enzyme activity [39]. The inhibitory effect of volatile oil against bacteria and fungi can be attributed to the higher content of biologically active compounds from the monoterpenes group, particularly terpinene, thymol, and carvacrol, where thymol has significant inhibitory activity against *S. aureus*, carvacrol and p-cymene against *Escherichia coli*, and γ-terpinene against *C. albicans* and *S. aureus* [17]. Thymol and carvacrol have increased activity against bacterial strains, while γ-terpinene and p-cymene are active against fungal strains [21,147]. The volatile oil extracted from *S. hortensis* L. has a broad antimicrobial spectrum, exhibiting inhibitory effects against 25 bacterial, eight fungal, and one yeast species [20]. Its activity against *E. coli*, *Salmonella typhimurium*, *S. aureus*, *Listeria monocytogenes*, and *Pseudomonas putida* isolated strains was also demonstrated [19]. The volatile oil has a higher concentration of antimicrobial compounds compared with the extracts [20,148].

The antioxidant activity of summer savory essential oil (SHEO) could be ascribed to the abundant content of carvacrol, γ-terpinene, p-cymene, and thymol compounds, which are known for their antioxidant properties [149,150]. Meanwhile, the components of extracts from *S. hortensis* (rosmarinic acid, caffeic acid, naringenin, quercetin, apigenin, kaempferol, luteolin, chlorogenic acid, rutin, and apigenin-glycoside) are also recognized for their antioxidant potential [151,152]. Due to their antioxidant activity, natural extracts derived from *S. hortensis* are being considered for use in the meat industry, with water leaf extract found to increase the shelf life of ground beef [153]. They can also be utilized as an antioxidant in mayonnaise formulations [154]. The presence of monoterpenes, such as carvacrol, cymene, and thymol, in the essential oil of *S. hortensis* suggests its potential for antimicrobial activities against food, plant, and human pathogens [155]. The antimicrobial mechanism involves damage to the integrity of the cell membrane, leading to the leakage of ions and other cell components and eventual death. At the same time, the antimicrobial properties of individual components of the essential oil are being evaluated [15].

The potential use of *S. hortensis* essential oil (SHEO) as a natural herbicide against two widely spread weeds, *Amaranthus retroflexus* and *Chenopodium album*, was also assessed [156]. The aerial parts of the plant were used during the fruit stage by hydro-distillation, and it was found to be rich in carvacrol and γ-terpinene (determined by GC-MS to be 55.66% and 31.98%, respectively). The essential oil was formulated as a nanoemulsion with a concentration of 5 mL/L, with an observed herbicidal activity both in laboratory conditions (at a nanoemulsion concentration of 1 mL/L) and in greenhouse conditions (at a nanoemulsion concentration of 4 mL/L).

There is a lack of data on the potential applications of *S. hortensis* essential oil in cancer treatment, although several other *Satureja* species have exhibited anticancer properties. For example, *S. intermedia* essential oil has shown potential against oesophageal squamous cell carcinoma and human bladder carcinoma cell lines, while *S. spicigera* has shown promise against Rectosigmoid adenocarcinoma cells, human epithelial colorectal adenocarcinoma cells, mouse embryo fibroblast cells, and ductal carcinoma cells. *S. sahendica* essential oil has demonstrated anticancer properties against breast cancer cells, fibroblast-like kidney cells, colon adenocarcinoma cells, and choriocarcinoma cells, while *S. montana* essential oil has shown potential against colon adenocarcinoma cells. These findings have been reported in various studies [17,155].

Some of the most important anatomical, physiological, and molecular changes of *S. hortensis* L. related to different stressors are presented in Table 4.

During stress periods, terpene emissions and related attracting mechanisms can indirectly contribute to plant defense mechanisms [164]. Furthermore, certain volatile compounds may act as airborne signals that can either directly or indirectly trigger systemic resistance and defense responses in neighboring plants [165,166].

Methyl jasmonate (MJ) treatment has been found to up-regulate genes involved in Jasmonate biosynthesis, secondary metabolism, and cell wall formation, as well as genes that encode stress-protective and defense proteins. Conversely, genes that are involved in photosynthesis, such as ribulose bisphosphate carboxylase/oxygenase, chlorophyll a/b-binding protein, and light-harvesting complex II, are down-regulated [167].

Gibberellin may have a potential role in aiding plants to adapt to stress [168,169]. Thus, the external application of it may reduce the negative impacts of salinity, while also enhancing growth under saline conditions, as evidenced by increased nutrient uptake, dry weights, plant height, leaf area, and yield, mitigating NaCl-induced growth inhibition [170,171]. Specifically, gibberellin application increased transpiration rate, relative water content, chlorophyll b, total chlorophyll, and xanthophyll content under salinity stress conditions for savory plants. The external application of gibberellin may enhance plant growth by elevating endogenous gibberellin levels [168]. Overall, gibberellin plays a crucial role in boosting plant growth, pigment synthesis, and photosynthesis rate under salt stress conditions [169].

In plants, the contents of hydrogen peroxide (H_2_O_2_) and malondialdehyde (MDA) reduced when inoculated with bacteria under well-watered and water stress conditions due to a significant increase in the expression of antioxidant enzymes, leading to a decline in MDA levels and electrolyte leakage [172]. Within plant cells, certain compounds such as lipid-soluble antioxidants (e.g., α-tocopherol and carotenoids), water-soluble reductants (e.g., glutathione and ascorbate), and antioxidant enzymes can provide protection against the harmful effects of ROS [173]. Moreover, the accumulation of certain osmolytes (e.g., proline) in plant cells can aid in scavenging free radicals and safeguarding enzymes [174].

### 3.9. Basil, Thyme, and Summer Savory Morphological Response to Different Biotic and Abiotic Stressors

All the three *Lamiaceae* herbs have developed several morphological adaptations that enable them to cope with biotic and abiotic stresses, such as leathery leaves, pubescence, and deep root systems.

Leathery leaves, one of the main adaptations, are characterized by a thick cuticle and more sclerenchyma tissue than other types of leaves, with a pronounced retaining water role, reducing water loss through transpiration, while conferring resistance to drought stress [175]. This adaptation allows these herbs to survive in arid conditions by maintaining hydration. Leathery leaves also help the plants to cope with high temperatures, which can cause dehydration and damage to the plant tissues. Moreover, the amount of epicuticular wax, a waxy substance found on the surface of leaves, was positively correlated with drought tolerance in these herbs [175]. The ability to tolerate water stress is linked to minor modifications in cellular biochemistry due to the buildup of compatible solutes and particular proteins that can be swiftly triggered by osmotic stress [176]. Water scarcity impacts plant development at diverse degrees, from the cell to the tissue level [177].

The presence of pubescence on the surface of these herbs’ leaves represents another significant mechanism of resistance (antixenosis) [175]. These tiny hairs act as a physical barrier against herbivores, such as insects and grazing animals, and reduce water loss through transpiration. They also help the plants to reflect some of the sunlight and reduce the amount of energy absorbed by the leaves, which protects them from photoinhibition or damage caused by excessive sunlight [175]. The phenylpropenes, along with terpenoids, are the primary components of essential oils that are released from the glandular trichomes of various *Lamiaceae* species [178]. In the particular case of basil, it predominantly synthesizes and stores eugenol and methyl chavicol in its glandular trichomes [179]. 

In addition, the three herbs have developed deep root systems, which enable them to access water and nutrients from deeper layers of soil and provide stability to the plant by anchoring it firmly in the ground. The deep roots allow the plants to survive in nutrient-poor soil and to withstand periods of drought stress by accessing water that is not available to shallower rooted plants [175]. Deep roots also provide stability to the plant by anchoring it firmly in the ground, protecting it from wind and water erosion. The root length and surface area of basil, thyme, and summer savory are positively correlated with their ability to cope with drought stress. In addition, a high root-to-shoot ratio, which is an adaptation that allows them to absorb water more efficiently and store it in their roots for future use, was observed [175]. 

### 3.10. Breeding Perspectives Regarding the Adaptability to the Main Abiotic Stressors

Genetic predisposition represents a fundamental requirement for enhancing the quality and yield of essential oils, with variety selection and plant breeding as additional factors. Induced polyploidization is one of the plant breeding techniques that can affect a plant’s genome, phenotype, physiology, and metabolome, enabling us to develop novel genotypes with better morphological, physiological, and biochemical properties.

Abiotic stressors such as drought, salinity, and extreme temperatures pose major challenges for herb cultivation. To address these challenges, research needs to focus on developing new cultivars of basil, thyme, and summer savory that are more resilient to abiotic stressors. One approach has been to identify genetic markers and traits associated with stress tolerance and breed for improved adaptability to stressful conditions. Studies have identified candidate genes and quantitative trait loci (QTLs) associated with drought tolerance in basil, thyme, and summer savory and used these genetic markers to develop new cultivars with improved drought resistance. 

Another promising approach has been to explore the use of plant growth-promoting rhizobacteria (PGPR) and other beneficial microorganisms to enhance the stress tolerance of these herbs. Recent studies have shown that the application of PGPR can improve the growth, yield, and quality of basil, thyme, and summer savory under stressful conditions and enhance their resistance to pests and diseases. 

In addition to phenotypic screening, molecular and biochemical approaches have been used to elucidate the mechanisms of stress tolerance in basil, thyme, and summer savory, as well as the identification of the key compounds involved. Numerous investigations have highlighted the significance of aquaporins in the plant stress response. TaTIP2;2 functions as an inhibitor of drought and salinity stress, its reaction not being reliant on ABA [180]. The transcriptomic and metabolomic responses of thyme plants to heat stress were investigated, and several candidate genes and metabolites associated with thermotolerance, including heat shock proteins, proline, and flavonoids, were identified [181]. Similarly, the volatile compounds and antioxidant activity of summer savory leaves under drought stress were analyzed, and the conclusions were that some volatile terpenes, such as γ-terpinene and carvacrol, were positively correlated with drought tolerance and antioxidant capacity [43].

To identify the compounds important for stress resistance in basil, thyme, and summer savory, several approaches have been used, such as metabolomics, transcriptomics, and proteomics. Analyses of basil leaves’ metabolites exposed to a water deficit found that the accumulation of phenolic acids and flavonoids was associated with drought tolerance [182]. Additionally, when the proteome of summer savory leaves under drought stress was investigated, it was found that the up-regulated proteins were related to photosynthesis, antioxidant defense, and stress response [183].

Regarding the germplasm availability, summer savory genotypes were evaluated for their tolerance to cold stress, and a promising candidate (“Mutika”) was identified, exhibiting better root volume, aerial part and total fresh weights, stem height, and flower number under low-temperature conditions [184].

### 3.11. Breeding Perspectives Regarding the Adaptability to the Main Biotic Stressors

Pests (aphids, thrips, spider mites, and whiteflies) are major biotic stressors that can affect basil, thyme, and summer savory crops. They feed on plant sap, causing stunted growth, leaf curling, and discoloration, together with a reduction in herb yield quantity and quality. Moreover, these pests can transmit viral diseases, such as tomato spotted wilt virus (TSWV), which can cause severe damage to the crops [185]. Breeding for pest-resistant cultivars using genetic markers could be a promising solution to reduce the use of insecticides and mitigate the effects of insect pests on the crops.

Diseases, such as fungal and bacterial infections, are also significant biotic stressors that can affect basil, thyme, and savory crops. The most common fungal diseases in these herbs include powdery mildew, downy mildew, and gray mold, which can cause leaf wilting, yellowing, and necrosis. Triggering defense genes against specific pathogens is influenced by distinct environmental factors. This indicates the involvement of intricate signaling pathways that empower plants to identify and safeguard against various stressors, including pathogenic threats [186,187]. Therefore, there is a need for breeding programs that focus on developing disease-resistant varieties of these crops. However, due to the limitations of traditional breeding, such as time-consuming and limited genetic diversity, new breeding technologies, such as genome editing, could provide an efficient and more precise method to introduce disease resistance into these crops [188]. 

Herbivores, such as deer, rabbits, and rodents, can also cause significant damage to basil, thyme, and savory crops. These herbivores can reduce crop yield by feeding on the plants and transmit plant diseases through their saliva [183]. Developing herbivore-resistant varieties of these crops can help reduce the impact of herbivores on crop production.

Weed competition is another important biotic stress that can affect the growth and yield of basil, thyme, and savory crops. Weeds compete with the herbs for nutrients, water, and sunlight, leading to reduced yield and quality of the crops. Furthermore, weeds can also act as hosts for pests and diseases, increasing their population and spread in the crop field. However, the excessive use of herbicides can have negative impacts on the environment and human health. Therefore, breeding for herbicide-resistant cultivars could be an important strategy in future breeding perspectives.

In terms of germplasm availability, when *Ocimum* accessions were screened for resistance to downy mildew caused by *Peronospora belbahrii*, the conclusion was that some genotypes, such as “Spice”, exhibited significantly lower disease severity and higher yield than others [189].

By overexpressing a gene encoding a chalcone synthase in basil plants, an increased resistance to Fusarium wilt caused by *F. oxysporum*, as well as higher levels of glyphosate-resistant basil and thyme flavonoids and phenolic acids were observed [190].

MYB and MYC proteins play a critical role in plants’ ability to cope with unfavorable environmental conditions. AtMYB30 functions as an activator of the hypersensitive cell death program upon pathogenic attack [191], while AtMYB33 and AtMYB101 are associated with ABA-mediated reactions to environmental cues [192]. AtMYB96 regulates water scarcity and disease resistance by acting through the ABA signaling pathway [193], and AtMYB15 is involved in enhancing cold stress tolerance [194].

Breeding for biotic stress resistance in basil, thyme, and savory crops is essential to ensure their sustainable cultivation and production. In recent years, there have been significant advancements in molecular breeding techniques, such as marker-assisted selection (MAS) and genomic selection (GS), which can accelerate the breeding process and improve the efficiency of selecting stress-tolerant traits [195]. Moreover, the identification of stress-responsive genes and pathways in these herbs can provide valuable targets for genetic engineering and biotechnological approaches to enhance their resistance to biotic stressors [192]. The breeding of basil with resistance to *F. oxysporum* has shown promising results [196].

Molecular markers and genetic engineering techniques can be used to accelerate the breeding process and identify genes responsible for biotic stress resistance in basil, thyme, and savory. Several resistance genes from basil (Pb1A and Pb1A′), which are responsible for resistance to downy mildew, a common fungal disease, were successfully transferred by researchers [197]. Similarly, genetic engineering can be used to develop herbicide-resistant cultivars.

## 4. Conclusions

Selecting and breeding basil, thyme, and summer savory genotypes with enhanced tolerance to biotic and abiotic stresses is crucial for their sustainable and profitable cultivation. Phenotypic screening, molecular and biochemical approaches, and genetic engineering or exogenous application of bioactive compounds are effective strategies for identifying and enhancing stress tolerance in these herbs. Identification of key compounds and genes involved in stress resistance can also provide valuable insights for further improvement of these important crops.

The research suggests that there is a significant potential for breeding and genetic improvement, as well as the use of microbial-based strategies, to enhance the adaptability of basil, thyme, and summer savory to abiotic stressors. These efforts could have important implications for the sustainability and productivity of herb cultivation in a changing climate and could help to ensure a reliable supply of these valuable herbs for food, medicinal, and other applications.

## Figures and Tables

**Table 1:** Main biocomponent profile of some *Lamiaceae* family aromatic plants presented by original research papers.

Aromatic Plant Species	Chemical Composition	Fresh Leaves	References
		Volatile Oil	
*O. basilicum* L.		Dry matter (909.1 g kg^−1^), cude ash (89.84 g kg^−1^), crude protein (208.8 g kg^−1^), ether extract (11.21 g kg^−1^), crude fiber (45.91 g kg^−1^), NFI (sugars readily hydrolyzed) (553.3 g kg^−1^), Mg (79.8 μg g^−1^), Ca (1278 μg g^−1^), K (2135 μg g^−1^), Na (218.5 μg g^−1^), Fe (26.31 μg g^−1^), Cu (1.95 μg g^−1^), Mn (8.56 μg g^−1^) and Zn (45.14 μg g^−1^) Alkaloids, tannins, flavonoids, cholesterol, terpernoids, glycosides, cardiac glycosides, phenols, carbohydrates, and phlobatannins	[8,9]
		(~6.20 mg/g) Linalool (56.7–60.6%), *epi*-α-cadinol (8.6–11.4%), α-bergamotene (7.4–9.2%) and γ-cadinene (3.2–5.4%, germacrene D (1.13.3%), camphor (1.13.1%)	[10,11]
*T. vulgaris* L.		Oxygen terpene derivatives (1,8-cineole, linalool, followed by camphor, endo-borneol, α-terpineol and linalyl acetate), terpene hydrocarbons (α-pinene, camphene and β–pinene, trans-caryophylle, four flavonoids (two flavanones and two flavones)—sakuranetin, 6,7-dimethylcarthamidin, respectively 5-desmethylsinensetin and -hydroxy-3,7,8,2′,4′-pentamethoxy-flavone	[12]
		(12 mL/kg ≤) Thymol (~47.59%), γ-Terpinene (~30.90%), para-Cymene (~8.41%), Carene<δ-2-> (~3.76%), Caryophyllene (2.68%), α-Thujene, α-Pinene, β-Pinene, β-Myrcene, α-Phellandrene, D-Limonene, β-Phellandrene, Terpineol, Terpinen-4-ol, Cyclohexene, 1-methyl-4-(5-methyl-1-methylene-4-hexenyl)	[13]
*S. hortensis* L.		Moisture (72%), protein (4.2%), fat (1.65%), sugar (4.45%), fibre (8.60%), ash (2.11%) Minerals: K (1.68–3.38 mg·kg−1 DM),P (0.31–0.72 mg·kg^−1^ DM), Ca (1.08–2.84 mg·kg^−1^ DM), Mg (0.25–0.61 mg·kg^−1^ DM), Fe (242–726 mg·kg^−1^ DM), and Na (0.007–0.013 mg·kg^−1^ DM)	[14,15] [16]
		(≥ 5%) Carvacrol (11–67%, Thymol (0.3–28.2%), γ-terpinene (15.30–39%), p-cymene (3.5–19.6%), α-phellandrene, α- and β-pinene, Sabinene, terpineol, α-thujene	[15,17], [18,19,20,21,22]

**Table 2:** *O. basilicum* L. response to different stressors.

Type of Stress		Anatomical, Physiological, and Molecular Changes	Contributing Factors	References
Abiotic	Salinity	Higher Na^+^ concentrations	Increased MDA accumulation	[64]
			Enhanced proline content	[4]
		Photosynthetic pigments decrease	Chlorophyllase enzyme activity enhancement	[65]
		Induces essential oil production	Higher oil gland density	[4,65,66]
	Drought	Plant growth process is inhibited	Constrained cell elongation and differentiation	[67,68]
		Disruption of main metabolic processes	Chlorophyll reduction	[69]
			Photosynthesis inhibation	[69]
			Cell division suppresion	[69]
		Protein complexes imbalance	Chlorophyll a and b depletion	[70,71]
		Chlorophyllase activity enhancement		
		Photosynthesis inhibition	Stomatal blockage	[72]
			RubisCO enzyme activity cut	[73]
		Cell osmotic adjustment	Proline accumulation	[69]
			CO_2_ assimilation	[74]
Biotic	Twospotted spider mite (*Tetranychus urticae* Koch)	Small chlorotic spots	Lower concentrations of nitrogen, phosphorous, and protein	[75]
		Cell physiology disruption	Photosynthesis reduction and phytotoxic compounds injection	[75,76]
		Plasma membrane potential change	Cytosolic free Ca^2+^ changes	[75]
		Oxidative damage	Increase in cellular concentration of reactive oxygen species and, subsequently, of H_2_O_2_	[75]

**Table 3:** *T. vulgaris* L. response to different stressors.

Type of Stress		Anatomical, Physiological, and Molecular Changes	Contributing Factors	References
Abiotic	Salinity	Nutritional imbalance in plant tissues	Electrolyte leakage increase	[103]
		Reduction of the photosynthetic capacity	CO_2_ assimilation reduction	[129]
	Drought	Mitigate cell division, elongation, and differentiation	Decreases cell turgor	[7,130]
			Minimizes enzyme activities	[7,130]
			Decreases energy supply	[7,130]
		Photosynthetic processes reduction	Lower level of relative water content (RWC)	[131,132]
		Affects the level of endogenous phytohormones	Alters relations between ABA, ethylene, GA3, cytokinins, and auxins	[7,133,134,135]
		Reduces concentration of chlorophyll a and b and total chlorophyll	Increases ROS production	[7,135,136,137]
		Carotenoids concentration reduction	Enhancement of ABA hormone	[7,133]
		H_2_O_2_ and lipid peroxidation enhancement	MDA concentration increase	[7]
		Adjustment of osmotic potential	Increased soluble sugars, proline, and free amino acid concentrations	[7]
		Considerable synthesis of total soluble phenols and phenylalanine ammonia-lyase (PAL)	Enhanced specific activity of PPO (polyphenol oxidase)	[7]
		Plant gene expression adjustment	Increased HDA-6 levels	[138,139,140]
		Secondary metabolites boost	Phe, Trp, and Asn amino acids	[141]
	Cd contamination (seeds)	Phytochelatin synthesis	Osmolytes (proline) accumulation	[142]
		ROS production enhancement	Increase in MDA content	[142]
Biotic	*Aphis serpylli* Koch	Cell physiology disruption	Carvacrol, Geraniol, and Thymol monoterpenes mitigate attack	[143,144]
			Linalol enhances attack	[143,144]

**Table 4:** *S. hortensis* L. response to different stressors.

Type of Stress		Anatomical, Physiological and Molecular Changes	Contributing Factors	References
Abiotic	Salinity	Growth decrese	High osmotic proficiency	[49,66]
			Salt ions toxicity	[66]
			Cytokinin cutoff	[66]
			Enhanced inhibitor production	[66]
			Decreasing water and nutrient uptake	[49]
		Higher Na^+^ concentrations	Lipid peroxidation increase	[157]
			Enhanced membrane damage	[157]
			Electrolyte leakage	[158]
			Increased MDA accumulation	[158]
			TPC, TSC, proline, and essential oil enhancement	[49]
		Chlorophyll content	Free oxygen radicals exposure/peroxidation	[49,159]
		Decreased transpiration rate	Gas exchange mittigation	[159]
		Imbalance in plant tissues	Reduced Ca and K	[49]
			Increased Cl and Na concentration	[49]
	Drought	Mitigate cell division, elongation, and differentiation	Decreases cell turgor	[160]
			Decreased relative water content (RWC)	
			Minimizes enzyme activities	[160]
			Decreases energy supply	[160]
			Reduced the plant height and the number of subsidiary branches	[43]
			Intensified malondialdehyde (MDA), H2O2, and proline contents	[43]
			Improved total chlorophyll, chlorophyll a and b, and carotenoid contents	[43]
Biotic	*Botrytis cinerea* Pers.	Necrosis and narrowing tissues	Endopolygalacturonase content enhancement	[161,162,163]
			Pectin degradation	

## Data Availability

Availability Statements are available in section “MDPI Research Data Policies” at https://www.mdpi.com/ethics accessed on 7 February 2023. No new data were created.
